# Optimal upper gastrointestinal surveillance strategies for gastric, small bowel and pancreatic cancer detection in Lynch syndrome

**DOI:** 10.1007/s10689-026-00567-y

**Published:** 2026-05-11

**Authors:** Tim Marwitz, Aleksander M. Bogdanski, Robert Hüneburg, Monique E. van Leerdam

**Affiliations:** 1 Department of Internal Medicine I, University Hospital Bonn, Bonn, Germany; 2 National Center for Hereditary Tumor Syndromes, University Hospital Bonn, Bonn, Germany; 3 Department of Gastroenterology and Hepatology, Leiden University Medical Center, Albinusdreef 2, 2333 ZA Leiden, The Netherlands; 4 Department of Gastrointestinal Oncology, Netherlands Cancer Institute, Amsterdam, The Netherlands

**Keywords:** Lynch syndrome, Small bowel cancer, Gastric cancer, Pancreatic cancer, Prevention, Endoscopy

## Abstract

Lynch syndrome is an autosomal dominant cancer predisposition syndrome and the most common cause of hereditary colorectal cancer. In addition to colorectal cancer, it confers substantially increased risks for several extracolonic malignancies. While there is broad consensus regarding the effectiveness of endoscopic colorectal surveillance, recommendations for surveillance of gastric, small bowel, and pancreatic cancers vary considerably among guidelines issued by leading professional societies. These discrepancies largely reflect the limited availability of robust, high-quality evidence. In this narrative review, we summarize the cancer risks for gastric, small bowel, and pancreatic malignancies in Lynch syndrome carriers, discuss recent studies evaluating the outcomes of surveillance strategies, and provide an overview of current guideline recommendations. Furthermore, we highlight emerging approaches that may enhance surveillance strategies in the future. In recent years, increasing research efforts have focused on surveillance for less frequent Lynch syndrome-associated malignancies, however, prospective data from large, well-characterized cohorts remain scarce. Such data are essential to harmonize existing guidelines and to enable the development of personalized surveillance strategies for individuals affected by Lynch syndrome.

## Introduction

Lynch syndrome (LS), the most common hereditary colorectal cancer (CRC) syndrome, results from germline pathogenic variants (PV) in mismatch repair (MMR) genes. While CRC and endometrial cancer dominate the clinical phenotype, LS confers elevated tumor risk across multiple extracolonic organs, including the upper gastrointestinal (UGI) tract and the pancreaticobiliary system [1, 2]. With improved CRC survival and the effectiveness of lower-gastrointestinal surveillance in reducing mortality, extracolonic malignancies now account for a growing proportion of the cancer burden in LS [3, 4].

Gastric cancer, small bowel cancer and pancreatic cancer are less common than CRC but carry high morbidity and often present at advanced stages. Early detection is challenging, and standardized surveillance strategies remain controversial. Retrospective cohorts, geographic variability, heterogeneous baseline risk, and inconsistent outcome reporting all complicate the development of evidence-based surveillance guidelines.

Despite these challenges, several considerations support the rationale for UGI surveillance in individuals with LS. Current guidelines uniformly endorse CRC surveillance in LS, given its proven efficacy in reducing CRC-related mortality. In contrast, the role of surveillance for other LS-associated malignancies, which occur at lower but still significantly increased rates compared with the general population, remains uncertain. Multiple studies have demonstrated elevated relative risk for gastric, small bowel and pancreatic cancer in carriers of PVs [2, 4–11]. A subset of these cancers is preceded by identifiable precursor lesions, including gastric and small-bowel adenomas, providing a window of opportunity for surveillance-based intervention. Advances in endoscopic and imaging techniques further enhance the feasibility of detecting and resecting lesions at early, potentially curable stages. Finally, accumulating evidence indicates substantial genotype-specific variation in UGI cancer risk, which is further modified by geographic background, sex and possibly a positive family history of UGI cancers.

In this article, we review available evidence on the risk of UGI malignancies in individuals with LS and critically assess data on the effectiveness of surveillance strategies. In addition, we provide an outlook on emerging technologies with the potential to enhance surveillance, including advances in imaging modalities, artificial intelligence (AI)-assisted detection, and biomarker-based risk stratification.

## Genotype-specific risk in gastric, small bowel, and pancreatic cancer

Rather than treating LS as a uniform condition, an increasing number of guidelines now emphasize its genetic heterogeneity. Large cohort studies have shown pronounced differences in cumulative UGI cancer risk between high-risk (*MLH1, MSH2* and *EPCAM*) and lower-risk (*MSH6* and *PMS2*) variants (Table 1) [1–4].

**Table 1:** Representative published cumulative risk estimates through age of 80 years for gastric, duodenal/small bowel, and pancreatic cancer in Lynch syndrome by gene* [6, 9, 11–14]

Gene	Gastric cancer	Duodenal/small bowel cancer^†^	Pancreatic cancer
*MLH1*	5–7%	0.4–11%	0–6.2%
*MSH2/EPCAM*^*‡*^	0.2–9%	1.1–10%	0.5–2.6%
*MSH6*	≤ 1–7.9%	≤ 1–4%	0–1.4%
*PMS2*	Near general-population risk/very rare	Very rare	0% in available cohort studies

*The age at which cumulative incidence is assessed varies across cohort studies, with estimates reported at ages 70, 75, or 80 years. For some studies, it is unclear whether participants were undergoing active surveillance, which may influence the reported risk estimates

^†^Some studies report cumulative incidence exclusively for duodenal cancer. In addition, risk stratification is in some cases limited to broad categories such as high- and low-risk variants

^‡^Data for *EPCAM* are sparse. Where reported, risk appears to parallel that of *MSH2* carriers

The lifetime risk of gastric cancer in the general population in Western countries is approximately 0.8% [12]. In contrast, individuals with LS have substantially higher gene-specific risks, with cumulative lifetime risk estimates of approximately 5–7% for *MLH1* carriers and a broader range of 0.2–9% reported for *MSH2* carriers [2, 6]. Data on *EPCAM* carriers are more limited due to the rarity of this PV, but available evidence suggests that gastric cancer risk parallels that observed in *MSH2* carriers [15–17]. For *MSH6* carriers, most cohort studies report low absolute risk, with cumulative incidence estimates generally below 2% by age 70, whereas *PMS2* carriers appear to have risk estimates that approximate those of the general population [1, 6–8, 18].

Small bowel cancer is a rare malignancy in the general population, with a lifetime risk estimated at approximately 0.3% [12]. As with gastric cancer, small bowel cancer risk in LS is strongly genotype dependent, with the highest cumulative incidence observed among carriers of *MLH1* and *MSH2* PVs ranging from 0.4 to 11% and 1.1 to 10% respectively [8, 13]. *MSH6* and *PMS2* carriers appear to have substantially lower risk, although precise penetrance estimates remain limited due to the rarity of events [13, 19, 20].

Although pancreatic ductal adenocarcinoma (PDAC) represents a recognized extracolonic manifestation of LS, gene-specific risk estimates remain imprecise. In the general population, the cumulative incidence of PDAC at age 80 is approximately 1%; this corresponds to about 0.7% by age 75 [11, 12]. In individuals with LS, cumulative incidence by age 75 appears highest among *MLH1* carriers (ranging from 1.4 to 6.2%), substantially lower in *MSH2* carriers (ranging from 0.5 to 2.1%), and minimal in *MSH6* and *PMS2* carriers [2, 3].

## Gastric cancer

### Epidemiology and risk factors

Large cohort studies consistently demonstrate that gastric cancer is part of the LS tumor spectrum, but that absolute risk estimates vary substantially by study design, population background, and case ascertainment. In the recent Dutch nationwide cohort study by Caspers and colleagues, gastric cancer was uncommon at younger ages and remained rare before age 50 (≤ 1% cumulative incidence across groups). However, risk increased thereafter, reaching a cumulative incidence of approximately 3% by age 70 and 5% by age 75 among high-risk PV carriers [6].

In an earlier cohort study from 2010, Capelle et al. evaluated 2014 LS mutation carriers from the Dutch Hereditary Cancer Registry and observed gastric cancer in 1.6% of carriers. Their Kaplan–Meier estimates suggested lifetime risks of 8.0% for men and 5.3% for women, with notable gene-specific differences (approximately 4.8% for *MLH1* and 9% for *MSH2* carriers), and no observed cases among *MSH6* carriers within that cohort. Most gastric cancers occurred in carriers without a family history, highlighting that family history alone is an imperfect discriminator of individual risk in LS [14].

More recently, community-based U.S. data further support sex-specific heterogeneity in risk estimates. In 1106 LS patients followed for a median of 19.3 years, Lin et al. identified 11 gastric cancers with a median age at diagnosis of 56 years. The cumulative incidence by age 80 was 7.26% in men and 3.43% in women. Gastric cancer risk was highest among *MSH2* and *MLH1* carriers, and a documented history of *Helicobacter pylori* (*H. pylori*) infection was associated with increased risk [8].

Surveillance-based cohorts, while not designed primarily for penetrance estimation, provide additional insight into clinical burden and age at detection in settings where upper endoscopy is routinely performed. In the German Consortium for Familial Intestinal Cancer registry, Ladigan-Badura et al. reported 49 gastric cancer events among 2009 individuals with LS. However, only a minority of cases could be included in analyses comparing surveillance-detected and symptom-detected cancers, due to incomplete documentation of surveillance history, symptoms, and tumor stage. Among the 22 patients with complete datasets, just six cancers were diagnosed under defined surveillance conditions, while 16 were detected after symptom onset. Within this limited subgroup, surveillance-detected cancers were more frequently diagnosed at an early stage than symptom-detected cancers, using tumor stage as a surrogate endpoint [18]. These findings suggest a potential stage shift associated with upper endoscopic surveillance but must be interpreted cautiously given the small number of evaluable surveillance cases, missing data for a substantial proportion of patients, and the absence of an intention-to-treat analysis or survival endpoints.

A recent systematic review identified male sex, age, high risk variants and *H. pylori*-infection as consistent risk factors [7]. This meta-analysis highlights substantial heterogeneity across studies, reflecting differences in study design, geographic setting, surveillance intensity, and mutation distribution. These findings reinforce conclusions from large population-based cohorts that gastric cancer risk in LS is neither uniform nor negligible and underscore the importance of gene-, sex-, and region-specific risk stratification when considering surveillance strategies.

#### Global and regional variation

Countries with a high incidence of gastric cancer in the general population such as Japan or Korea, also demonstrate a higher baseline prevalence among LS carriers, with a reported cumulative risk of up to 41% by the age of 70 years, as well as an increased risk of synchronous and metachronous gastric cancer compared with Western countries [6, 14, 21–23].

The underlying reasons for this disparity remain incompletely understood. As most of those gastric cancers described in the studies exhibit a high frequency of microsatellite instability or MMR-deficiency, a hallmark for LS-associated malignancy, this geographic heterogeneity is thought to reflect interactions with other genetic predispositions, environmental factors and *H. pylori* incidence [21, 23, 24].

Although the available data are based on relatively small cohorts, some authors have suggested more intensive surveillance strategies, particularly for LS carriers with a prior diagnosis of gastric cancer [23]. The NCCN guidelines partially incorporate these findings, recommending that LS carriers residing in or originating from high-incidence regions, such as Asia, may be considered for earlier initiation of UGI surveillance [13].

#### Influence of *Helicobacter pylori*, autoimmune gastritis, and family history

According to the Lauren classification, gastric cancer comprises two major histological entities, intestinal-type gastric cancer and diffuse-type gastric cancer, which are characterized by distinct pathogenetic mechanisms [25]. Intestinal-type gastric cancer typically develops through the Correa cascade, a multistep process describing the progression from chronic gastritis to multifocal atrophic gastritis, intestinal metaplasia, dysplasia, and ultimately carcinoma, and is frequently associated with *H. pylori* infection or autoimmune gastritis [26].

By contrast, diffuse gastric cancer generally lacks recognizable precursor lesions and arises from poorly cohesive tumor cells that infiltrate the gastric wall diffusely, often as a consequence of disrupted cell-to-cell adhesion. This histological subtype is less strongly linked to chronic gastritis or intestinal metaplasia and is associated with distinct molecular alterations affecting epithelial integrity [27].

Obesity, smoking, alcohol consumption, and *H. pylori* infection represent the most significant modifiable risk factors for gastric cancer in the general population, with the strongest associations observed for intestinal-type gastric cancer [24, 27]. Notably, substantial racial and ethnic disparities in the incidence of diffuse-type gastric cancer have been reported, with Asian, Hispanic White, and Native American populations demonstrating significantly higher incidence compared with non-Hispanic White individuals [27, 28].

Despite limited data, cohort studies indicate that intestinal-type gastric cancer predominates in LS (57–79%), with diffuse-type gastric cancer accounting for 17–29% of cases [6, 14, 15, 29]. Among LS carriers, the prevalence of *H. pylori* infection appears comparable to that observed in the general population, and some studies have demonstrated an association between *H. pylori* infection and the detection of gastric cancer or precancerous lesions during surveillance endoscopy [30, 31]. In contrast to the general population, where families with multiple gastric cancer cases often exhibit increased *H. pylori* prevalence, no such familial clustering of *H. pylori* infection has been demonstrated in LS [30, 32]. Even though the role of *H. pylori* in LS-associated gastric carcinogenesis remains unclear, the increased gastric cancer incidence observed among PV carriers living in regions with high *H. pylori* prevalence suggests that environmental exposure may act as an additional modifying risk factor [22].

Pyloric gland adenomas and gastric tubular adenomas exhibiting microsatellite instability (MSI) have been described as a rare but potential precursor lesion in LS patients raising the possibility of an alternative pathway to gastric carcinogenesis [33–35]. This observation is consistent with a potentially accelerated progression from adenoma to adenocarcinoma in LS patients [19].

A molecular classification of gastric cancer proposed by The Cancer Genome Atlas Program recognizes MSI-high tumors as a distinct subtype characterized by hypermutation. Such tumors may develop sporadically, most frequently due to epigenetic silencing of *MLH1*, or occur in the setting of LS as a consequence of germline mismatch repair deficiency [36].

The influence of family history on gastric cancer risk in LS remains incompletely understood. Recent data from a large U.S. cohort indicate that individual risk increases with each affected first-degree relative [34, 37]. In contrast, this association was not observed in another study, in which 68% of patients diagnosed with gastric cancer reported no family history [18]. These findings imply that family history may serve as an additional risk stratification in selected patients, although its role appears less pronounced and less consistent than in the general population.

### Surveillance strategies

#### Endoscopy: intervals and biopsy protocols

The role of esophagogastroduodenoscopy (EGD) in the management of LS remains incompletely defined, and recommendations from professional societies differ substantially [13, 38–43]. While some guidelines advocate risk-adapted surveillance, others discourage routine EGD in the absence of additional risk factors, reflecting ongoing uncertainty regarding the balance of benefit, burden, and resource utilization (Table 2).

**Table 2:** Recommendation of gastric cancer surveillance and *Helicobacter pylori* (*H. pylori*) testing in current guidelines [13, 38–40, 42, 43]

Guideline/organization	Date of publication/last update	Recommendation for esophagogastroduodenoscopy (EGD) surveillance	Suggested starting age	Suggested interval	*H. pylori* testing and treatment
NCCN (National Comprehensive Cancer Network, US)	2025	Recommend EGD surveillance except for *PMS2*	30–40 years	Every 2–4 years, < 2 years if high risk	Recommend screening for *H. pylori*
ACG (American College of Gastroenterology)	2015	Consider EGD in those with family history of gastric/duodenal cancer or high-incidence background	30–35 years	Every 3–5 years when risk factors present	*H. pylori* testing and treatment advised
ESGE (European Society of Gastrointestinal Endoscopy)	2019	Do not recommend routine EGD			Recommend non-invasive *H. pylori* testing and treatment
EHTG (European Hereditary Tumour Group)	2020	Do not recommend routine EGD			Recommend screening for *H. pylori*
DGVS (German Society for Gastroenterology, Digestive and Metabolic Diseases)	2025	Recommend EGD surveillance, consider in *PMS2*	30 years	Every 1–4 years	*H. pylori* testing and treatment advised
BSG (British Society of Gastroenterology)	2019	Do not recommend routine EGD			*H. pylori* testing and treatment advised

Robust data demonstrating an impact of EGD surveillance on clinically meaningful outcomes, including cancer-specific mortality, are currently lacking. Similarly, comprehensive cost-effectiveness analyses remain scarce, particularly outside selected high-risk subgroups.

Uncertainty is further compounded by incomplete knowledge regarding the natural history of gastric precursor lesions. The risk of progression from intestinal metaplasia or other premalignant conditions to invasive cancer has not been well quantified in PV carriers, and available data are largely extrapolated from sporadic gastric cancer cohorts [6, 31, 34]. This gap limits the ability to define which lesions warrant intensified surveillance or intervention.

One of the largest surveillance studies evaluating UGI endoscopy in LS reported a gastric cancer detection rate of approximately 0.39% per EGD examination [18]. Although the absolute yield appears low, most detected gastric cancers were identified at an early stage, consistent with a potential stage shift attributable to surveillance [19]. Other studies have shown that upper endoscopy yields clinically actionable findings in about 16–17.6% of individuals. These findings mainly include non-dysplastic, but also dysplastic, lesions of the UGI tract, with the highest detection rates observed during the first two examinations [35, 44].

To date, beyond carriers of high-risk PVs, it remains unclear which additional subgroups, defined by age, sex, geographic background, or family history, derive the greatest benefit from regular EGD surveillance.

There is currently no data supporting evidence-based surveillance intervals for UGI endoscopy in LS. Existing guidelines that recommend EGD surveillance propose intervals ranging from 1 to 5 years, reflecting expert opinion rather than empirical evidence. Given the well-established accelerated adenoma-carcinoma sequence in LS-associated CRC, it has been hypothesized that a similarly rapid progression may occur in the UGI tract, which would favor shorter surveillance intervals in selected high-risk individuals. However, this assumption remains unproven and underscores the need for prospective studies.

There are relatively few studies specifically addressing endoscopic quality parameters during UGI endoscopy in patients with LS. In an international expert statement, Farha and colleagues proposed a standardized, high-quality approach to EGD surveillance in this population, emphasizing a careful mucosal inspection lasting a minimum of seven minutes using high-definition white-light endoscopy, systematic non-targeted biopsies following the Sidney protocol, and resection of gastric polyps measuring more than 10 mm in diameter. However, it should be noted that most of these quality markers were established through consensus-based expert opinion rather than evidence derived from prospective outcome studies in LS-specific cohorts [45]. Several of these parameters, including prolonged inspection time and systematic examination of the gastric mucosa, have been associated with improved neoplasia detection rates in the general population [46]. Nevertheless, their applicability to individuals with LS remains uncertain, as LS-specific data are lacking.

While the development of standardized EGD protocols and quality indicators in LS is desirable to improve surveillance quality and comparability across centers, key parameters such as inspection time, systematic photo documentation of anatomical landmarks (including the ampullary region), and the use of deep sedation have not yet been validated with respect to clinically relevant outcomes in this population. As such, these measures should currently be regarded as hypothesis-generating rather than evidence-based standards, highlighting the need for prospective studies to determine whether they translate into improved detection rates and patient-relevant outcomes in LS.

#### *Helicobacter pylori* testing and eradication

Population-based studies have demonstrated that *H. pylori* screening and eradication constitute a cost-effective strategy for gastric cancer prevention, even in countries with relatively low baseline prevalence of infection [47]. Usui et al. demonstrated that carriers of PVs in cancer-predisposing genes with *H. pylori* infection had a significantly higher cumulative risk of gastric cancer compared with *H. pylori*-infected noncarriers [48]. Accordingly, most professional society guidelines, including those that do not recommend routine endoscopic surveillance, advocate testing for and treating *H. pylori* infection as a preventive measure [38–40, 49, 50].

As most guidelines advocate *H. pylori* testing, non-targeted biopsies following the Sidney protocol are commonly recommended as part of a high-quality baseline examination. In the absence of endoscopy, validated non-invasive diagnostic modalities, such as urea breath testing or stool antigen testing, are recommended alternatives.

To date, direct evidence evaluating gastric cancer-related outcomes following *H. pylori* eradication in LS carriers is lacking. Nevertheless, eradication therapy is generally assumed to confer a protective effect comparable to that observed in the general population, given the established causal relationship between *H. pylori* infection and gastric carcinogenesis.

### Emerging approaches in gastric cancer surveillance

#### Image enhancement and artificial intelligence-assisted endoscopy

High-definition endoscopy, in combination with image-enhanced endoscopic technologies, has been shown to improve the detection and characterization of early gastric cancer in the general population [51, 52]. Image-enhanced modalities, such as narrow-band imaging and linked color imaging may facilitate the identification of subtle lesions that are difficult to detect using white-light endoscopy alone. As virtual chromoendoscopy becomes increasingly integrated into routine endoscopic practice, its use in surveillance settings is expected to expand.

AI-based systems for the UGI tract have not yet been implemented in routine clinical practice but already demonstrate considerable promise for the detection of early gastric cancer, with high sensitivity reported in retrospective and prospective validation studies [53, 54]. Although these systems have not been specifically validated in LS populations, AI-assisted endoscopy may eventually support real-time identification of early flat lesions and characteristic intestinal metaplasia patterns, thereby assisting endoscopists in targeted biopsy acquisition.

#### Serologic and molecular markers

Human pepsinogens are inactive precursors of pepsin secreted by gastric glands, with pepsinogen I predominantly produced in the fundic mucosa and pepsinogen II secreted by both fundic and antral glands. Because atrophic gastritis, most commonly related to *H. pylori* infection, typically begins in the antrum and progresses proximally toward the corpus, the serum pepsinogen I/II ratio has been proposed as a surrogate marker of gastric mucosal atrophy and therefore gastric cancer risk. While less invasive strategies may offer improved acceptability among the general population, their reported accuracy (Sensitivity of 57% in a recent meta-analysis) is currently inadequate to justify implementation as surveillance tests [55–58].

Another emerging approach for non-invasive cancer detection involves circulating microRNAs, which are aberrantly expressed in tumor tissue and can be detected in peripheral blood. Several studies have demonstrated the potential of microRNA panels to identify gastric cancer in high-risk populations with promising diagnostic performance [59]. Further research is necessary to determine the usefulness in LS individuals.

While serologic and molecular biomarkers have been evaluated primarily in sporadic gastric cancer or high-risk populations, data on their performance in LS carriers are currently lacking. Nevertheless, such biomarkers may eventually enable non-invasive risk stratification or facilitate early cancer detection in individuals who are not candidates for regular endoscopic surveillance.

## Small bowel cancer in Lynch syndrome

### Epidemiology, risk factors and lesion distribution

#### Incidence and clinical relevance

Small bowel cancer is rare in the general population but occurs with markedly increased frequency in individuals with LS [5, 60, 61]. Prospective and registry-based data estimate a lifetime penetrance ranging from approximately 3–11% among PV carriers, confirming small bowel cancer as a clinically relevant extracolonic malignancy in this population [17]. A population-based Spanish study further demonstrated that LS accounts for a substantial proportion of small bowel cancer cases, with pathogenic MMR variants identified in approximately 10% of all patients diagnosed with small bowel cancer [62]. Furthermore, the risk of duodenal cancer is much higher among individuals with LS compared to the general Dutch population with a standardized incidence ratio of 250 (95% CI 178–290) [6].

In a recent German multicenter study evaluating UGI endoscopic detection of duodenal cancer in LS, the median age at diagnosis was 51.7 years; however, notably, approximately 10% of affected individuals were diagnosed before the age of 35, underscoring the potential for early-onset disease in selected carriers [20]. In contrast, among 1861 LS carriers from the Dutch national LS database, no-one was diagnosed with duodenal cancer before the age of 40 years [6]. Jejunal and ileum cancers were not included in this analysis.

These findings highlight the clinical relevance of small bowel cancer despite its overall rarity, as early-onset presentation and diagnostic delay may contribute to adverse outcomes, particularly given the nonspecific symptoms and limited accessibility of the small bowel to routine endoscopic evaluation.

To date, family history does not appear to be a strong or consistent predictor of small bowel cancer occurrence in LS, with several studies failing to demonstrate a clear association between affected relatives and individual cancer risk [20].

#### Lesion distribution

**Fig. 1:**
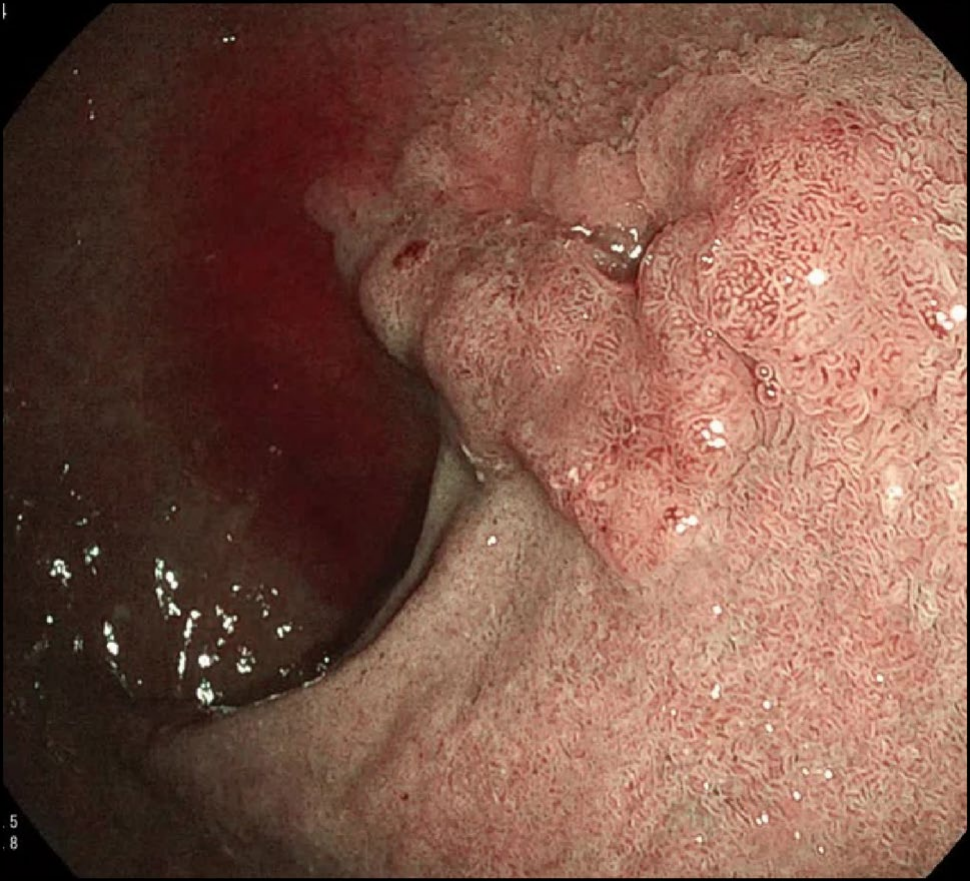
Adenocarcinoma in the duodenal bulb of a patient with a pathogenic variant in *MSH2*

Data on the exact anatomical distribution in LS remains limited. Available evidence suggests that tumors are most frequently located in the proximal small bowel. In a meta-analysis, small bowel cancers were predominantly identified in the duodenum (43%), followed by the jejunum (37%) and the ileum (20%) [63]. This proximal predominance has important clinical implications, as duodenal and proximal jejunal lesions are more amenable to detection by UGI endoscopy or device-assisted enteroscopy than distal tumors (Fig. 1). The observed distribution pattern may partly explain the relatively higher detection rates of duodenal cancers in surveillance-based cohorts.

#### Risk factors

The presence of duodenal adenomas has been proposed as a potential marker of increased small bowel cancer susceptibility. Duodenal adenomas may reflect a more generalized predisposition to neoplasia throughout the small bowel and have been associated with an increased likelihood of synchronous or metachronous neoplastic lesions [20, 31]

Additional factors associated with increased small bowel cancer risk include male sex and a personal history of other LS-associated gastrointestinal neoplasms [2, 20]. However, given the low absolute incidence, the independent contribution of these factors remains difficult to quantify, and robust risk models incorporating genotype, clinical history, and lesion characteristics are currently lacking (Fig. 1).

### Surveillance strategies

#### Esophagogastroduodenoscopy (EGD)/colonoscopy

As most small bowel cancer occurs in the duodenum, EGD as standard surveillance seems feasible [19, 20, 61, 64]. Push enteroscopy (PE) offers visualization of the distal duodenum and proximal to mid jejunum and can be performed in conjunction with standard EGD with minimal additional effort by using a longer scope. Given that some studies have shown that approximately one-third of duodenal lesions are beyond the reach of conventional EGD, PE may be considered a safe supplemental technique [61]. However, to date, no published data demonstrates a clear clinical benefit of PE as a routine surveillance tool in LS, and earlier endorsements in some guidelines have subsequently been removed [64].

As ileal carcinoma accounts for only approximately 20% of small bowel cancers, the clinical value of routine intubation of the terminal ileum during colonic surveillance remains uncertain. Future studies are needed to determine whether systematic visualization of the terminal ileum improves cancer detection or clinical outcomes in this setting.

#### Capsule endoscopy (CE)

Capsule endoscopy (CE) is a minimally invasive modality that allows visualization of the entire small bowel and is generally well tolerated, with a low complication rate [65]. Despite its advantages, CE may fail to detect even larger small bowel lesions, particularly those with flat morphology [66, 67]. In a prospective cohort, Perrod et al. reported a diagnostic yield for small bowel adenomas and carcinomas of 4.4% at the first CE and 4.6% at a second examination performed 2 years later [68]. In contrast, a recent meta-analysis demonstrated substantially lower diagnostic yield after histological confirmation, with detection rates dropping to 0% on repeat CE examinations [69]. High miss rates for advanced small bowel neoplasia reported with CE in the general population, the need for additional invasive endoscopic evaluation when a suspected lesion is detected, and its suboptimal performance in LS cohorts argue against its use as a surveillance modality in patients with LS [70].

#### Magnetic resonance enterography (MRE) and computed tomographic enterography (CTE)

Magnetic resonance enterography (MRE) and computed tomographic enterography (CTE) provide cross-sectional imaging of both the small bowel lumen and wall, enabling detection of mural masses and extraluminal disease extension. In patients with Peutz-Jeghers syndrome, MRE has been shown to be less sensitive than CE for the detection of small bowel polyps [71]. Although CT enterography (CTE) appears to be more sensitive than capsule endoscopy (CE) for detecting small-bowel lesions in the general population, this observation has not been confirmed in the limited data available from LS cohorts [70, 72].

Given their ability to detect transmural disease and extraluminal involvement, MRE and CTE may play a complementary role in the diagnostic workup of suspected small bowel malignancy or in the evaluation of symptoms suggestive of advanced disease, particularly in patients in whom CE is contraindicated due to concerns regarding capsule retention.

Although data specifically evaluating MRE and CTE in LS are sparse, available evidence suggests that they have limited utility as a primary surveillance modality for small bowel cancer in this population.

#### Device-assisted enteroscopy

Device-assisted enteroscopy (DAE), including double-balloon enteroscopy, allows endoscopic visualization of large portions of the small bowel and enables therapeutic interventions such as biopsy and polypectomy. Although complication rates are generally low and diagnostic success is high when targeting known lesions, DAE is time-consuming and invasive and places a considerable operational burden on endoscopy units [73]. As a result, DAE is not considered suitable for routine surveillance but is best reserved for diagnostic clarification or therapeutic intervention following lesion detection by other modalities especially CE.

#### Value of small bowel surveillance

Although small intestine cancer in LS is associated with a 5-year survival rate of about 67%, suggesting a potential role for surveillance, its low incidence, particularly in lower-risk genotypes, does not currently support routine screening [74]. Large prospective studies are needed to clarify whether targeted surveillance is justified in high-risk subgroups and to define the optimal modality, starting age, and surveillance intervals. In the meantime, LS carriers should be routinely evaluated for suggestive symptoms and, if present, undergo diagnostic assessment with CE, MRE or CTE.

Taken together, the available evidence suggests that while duodenal lesions may be incidentally detected during routine EGD, there is currently no sufficient basis to recommend structured small bowel surveillance in LS. Future prospective studies are required to determine whether targeted approaches in selected high-risk subgroups may be justified.

### Emerging approaches in small bowel surveillance

AI-assisted image interpretation in radiology represents a rapidly evolving area of research with potential relevance for small bowel surveillance. Machine learning-based approaches have already demonstrated utility in the diagnosis of small bowel Crohn’s disease using MRE and in differentiating benign from premalignant colorectal polyps detected by CT colonography [75, 76]. These technologies may ultimately contribute to improved lesion detection and reduced interobserver variability in imaging-based surveillance.

AI also holds promise for CE interpretation. Several studies have demonstrated that AI-assisted CE reading can significantly increase diagnostic yield while simultaneously reducing reading time, thereby addressing one of the major practical limitations of capsule-based surveillance [77, 78]. Such approaches may be particularly relevant in LS, where lesion prevalence is low and subtle mucosal abnormalities may be overlooked during conventional manual review, although dedicated validation in this population remains necessary.

Liquid biopsy-based approaches aimed at assessing microsatellite instability (MSI) status are an emerging area of investigation in LS. Early studies suggest that circulating tumor-derived DNA may allow detection of neoplastic and potentially preneoplastic lesions in MMR-deficient individuals. However, current data remain preliminary, and available assay platforms demonstrate suboptimal sensitivity and specificity, limiting their immediate applicability for small bowel cancer screening [79].

In parallel, methylated DNA marker panels have shown high accuracy in distinguishing CRC and endometrial cancer from benign tissue in LS, but remain far from routine clinical implementation [80]. Similar strategies may, in the future, be adapted for small bowel carcinoma detection.

## Pancreatic cancer

### Epidemiology and risk factors

#### Incidence estimates for pancreatic cancer

PDAC is a recognized but relatively uncommon component of the LS tumor spectrum. Across published studies, estimated lifetime risks vary, reflecting heterogeneity in cohort composition, outcome definitions, gene stratification and follow-up duration. Most studies report cumulative incidence up to age 70, with estimates ranging from 0 to 3.9%, depending on the mismatch repair gene involved [2, 4, 9–11]. Key studies are summarized below and an overview is provided in Table 3.

**Table 3:** Published estimates of pancreatic cancer risk in Lynch syndrome genes (Bogdanski et al. report pancreatic ductal adenocarcinoma (PDAC) risk only) [2, 4, 9–11]

Study	Sample size	Age at estimate	MLH1	MSH2/EPCAM	MSH6	PMS2
Barrow et al. [4]	839	70	0%	0.7% (0.0–1.4)	0%	–
Kastrinos et al. [10]	6342	70	3.7% (1.5–5.9) in the overall cohort			
Møller et al. [2]	3119	70	3.9% (1.4–6.4)	0.5% (0.0–1.5)	1.4% (0.0–4.2)	0%
		75	6.2% (2.6–9.8)	0.5% (0.0–1.5)	1.4% (0.0–4.2)	0%
Bujanda et al. [9]	425	70	3.3% (0.0–7.0)	2.6% (0.0–7.7)	0%	0%
Bogdanski et al. [81]	2605	70	0.4% (0.1–1.8)	1.3% (0.6–2.8)	0.2% (0.0–1.3)	0%
		75	1.4% (0.5–4.0)	2.1% (1.0–4.1)	0.2% (0.0–1.3)	0%

More recently, a prospective nationwide Dutch cohort of 2605 individuals with LS was analyzed [11]. By age 70, PDAC-specific cumulative incidence was low across all gene groups, estimated at 0.4% (95% CI 0.1–1.8) for *MLH1*, 1.3% (95% CI 0.6–2.8) for *MSH2/EPCAM*, 0.2% (95% CI 0.0–1.3) for *MSH6*, and 0% for *PMS2* carriers. When follow-up was extended to age 75, cumulative PDAC incidence increased to 1.4% (95% CI 0.5–4.0) among *MLH1* carriers and 2.1% (95% CI 1.0–4.1) among *MSH2/EPCAM* carriers, while remaining unchanged for *MSH6* and *PMS2* carriers. By age 75, the relative risks compared with the general population were 2.0 (95% CI 0.7–5.7) for *MLH1*, 3.0 (95% CI 1.5–6.1) for *MSH2/EPCAM* carriers, 0.3 (95% CI 0.0–Inf) for *MSH6* carriers and 0 for *PMS2* carriers. Importantly, from a surveillance perspective, pancreatic imaging is not specific to PDAC and may also detect other malignancies arising in the periampullary region. Accordingly, outcome definitions were expanded to include anatomically related pancreaticobiliary malignancies, namely ampullary carcinoma and distal cholangiocarcinoma. When considering all malignancies detectable through pancreatic imaging by MRI or EUS (PDAC, ampullary carcinoma and distal cholangiocarcinoma), the cumulative incidence by age 75 was higher, reaching 3.0% (95% CI 1.5–5.8) among *MLH1* carriers, 3.4% (95% CI 2.0–5.8) among *MSH2/EPCAM* carriers, and 1.0% (95% CI 0.3–2.7) among *MSH6* carriers, with no events observed among *PMS2* carriers. It is important to note the distinction when interpreting the literature. Several earlier studies defined pancreatic cancer broadly, including pancreatic neuroendocrine tumors, which have a more favorable prognosis (5-year survival of 48%) than PDAC [82]. Although PDAC represents 90% of all PCs, even one or more additional non-PDAC events can meaningfully increase cumulative incidence in cohorts with few events [83]. In contrast, the Dutch cohort study restricted outcomes to PDAC because of its poor prognosis and relevance for surveillance, yielding more specific but potentially lower risk estimates than studies that combined all PC subtypes.

In a large international cohort of 3119 LS carriers, Møller et al. evaluated the risk of multiple LS-associated malignancies, including pancreatic cancer [2]. By age 70, PC-specific cumulative incidence was highest among *MLH1* carriers (3.9%; 95% CI 1.4–6.4). Substantially lower risks were observed among *MSH2* carriers (0.5%; 95% CI 0.0–1.5) and *MSH6* carriers (1.4%; 95% CI 0.0–4.2). No cases were observed in *PMS2* carriers. When follow-up was extended to age 75, cumulative incidence increased to 6.2% (95% CI 2.6–9.8) among *MLH1* carriers, while the estimates for other PV carriers remained unchanged. Despite higher point estimates, the 95% CIs overlap with the Dutch cohort study, indicating no significant difference.

A Spanish multicenter study by Bujanda et al., including 425 LS PV carriers, reported comparable gene-specific patterns [9]. By age 70, PC risk was elevated among *MLH1* carriers (3.3%; 95% CI 0.0–7.0) and *MSH2* carriers (2.6%; 95% CI 0.0–7.7), with no observed PC cases in *MSH6* or *PMS2* carriers. In addition to PC, this study evaluated cholangiocarcinomas and reported increased risks by age 70 among *MLH1* (8.8%; 95% CI 0.8–14.3) and *MSH2* carriers (1.4%; 95% CI 0.0–4.1). Underscoring that these individuals are also at risk for other pancreaticobiliary tumors. It is important to note that while a distinction was made between intrahepatic and extrahepatic cholangiocarcinomas, the exact anatomic location was not specified.

Earlier studies have reported heterogeneous estimates of PC risk in LS and are often constrained by methodological limitations. In a large retrospective cohort of 6342 LS carriers, Kastrinos et al. reported a cumulative PC risk of 3.7% (95% CI 1.5–5.9) by age 70 [10]. However, analyses were not stratified by gene and the majority of PC diagnoses lacked both clinical and pathological verification. Barrow et al. studying 839 individuals, reported a cumulative PC incidence by age 70 of 0.7% (95% CI 0.0–1.4) among *MSH2* carriers and observed no PC cases among *MLH1* or *MSH6* carriers [4]. Interpretation of these findings is limited by inclusion of individuals with genetically unconfirmed LS. To obtain more accurate and personalized incidence estimates, additional data are needed, with stratification by gene, age and ideally incorporating other potential risk factors such as socioeconomic status and lifestyle.

#### Role of family history

Current surveillance recommendations consider individuals with LS and a positive family history eligible for PDAC surveillance [40, 43, 84, 85]. However, empirical evidence supporting an association between family history of PDAC and increased PDAC risk among LS carriers is limited. To date, only two studies have evaluated this relationship. In the Spanish multicenter cohort reported by Bujanda et al., one of four individuals diagnosed with PC had a reported positive family history [9]. In contrast, in the national Dutch cohort no familial clustering of PDAC was observed [11].

It is important to acknowledge potential limitations in the assessment of family history. In earlier generations of families with LS competing mortality from colorectal cancer may have limited the opportunity for PDAC to manifest [86]. In addition, historical underdiagnosis or incomplete documentation of PDAC could have obscured familial clustering. As advances in cancer prevention, surveillance and treatment continue to improve survival from colorectal cancer, PDAC incidence and familial aggregation may become more apparent [86].

Overall, current evidence does not support family history of PDAC as an independent determinant of surveillance eligibility among individuals with LS. Further longitudinal studies with systematic assessment of family history will be required to clarify whether it modifies PDAC risk in this population.

### Surveillance strategy recommendations and evidence

#### Surveillance eligibility and recommendations

Eligibility for PDAC surveillance is based on an arbitrary lifetime PDAC risk threshold of 5%, which is derived from expert consensus [87]. Importantly, estimates of lifetime risk depend on the age up to which cumulative incidence is calculated. While many older studies report risk through age 70, this cutoff may underestimate clinically relevant risk, as PDAC surveillance may be considered until age 75. Conversely, extending risk estimates beyond this age is unlikely to be clinically meaningful because of competing mortality and diminishing benefit, although no uniform stopping age for surveillance has been established and decisions may vary between individuals especially with the increase of a vital elderly population [87]. Interestingly, PDAC surveillance modalities are not specific to PDAC and may also detect anatomically related pancreaticobiliary malignancies, such as ampullary carcinoma and distal cholangiocarcinoma, which could also benefit from early detection through pancreatic imaging. Consideration of these malignancies in addition to PDAC may therefore be relevant when assessing whether lifetime risk thresholds for surveillance eligibility are met.

Guideline recommendations on PDAC surveillance in individuals with LS are heterogeneous and reflect uncertainty about absolute risk and clinical benefit of surveillance [40, 43, 84, 85]. The NCCN guidelines recommend consideration of PDAC surveillance in LS carriers with a positive family history of PDAC [84]. Similarly, international expert consensus statements, such as those from the International Cancer of the Pancreas Screening (CAPS) Consortium support PDAC surveillance in LS carriers with a family history with PDAC [87]. In contrast, several other guidelines do not recommend routine PDAC surveillance for individuals with LS. The British Society of Gastroenterology, the European Hereditary Tumor Group/European Society of Coloproctology, and the InSiGHT group do not endorse routine PDAC surveillance in LS but note that surveillance may be considered within clinical trials or in LS carriers with a strong family history of PDAC [43, 88]. The European Society of Gastrointestinal Endoscopy (ESGE) does not provide a specific recommendation for PDAC surveillance in LS [85]. Where offered, PDAC surveillance is conducted in specialized expert centers and consists of annual MRI, annual EUS or a combination of both [89]. In the presence of concerning lesions, surveillance intervals may be shortened or further evaluation with EUS-guided biopsies may be pursued [87, 89].

#### Available evidence

To date, data about outcome of PDAC surveillance in LS are lacking. Across published surveillance studies, approximately 80 LS carriers have been reported to undergo PDAC surveillance. The largest contribution comes from the prospective study by Dbouk et al., which included 58 LS carriers [90]. The remaining individuals were reported across smaller surveillance cohorts, as summarized in a prior review [89]. No cases of PDAC have been reported among LS carriers undergoing surveillance to date [89]. However, interpretation is limited by incomplete reporting of gene distribution, age and follow-up time, making it difficult to determine whether the absence of detected PDAC reflects low underlying cancer risk or insufficient sample size.

Evidence supporting benefit of PDAC surveillance is largely based on other high-risk populations. In a large multicenter study including predominantly individuals from familial pancreatic cancer (FPC) kindreds (981 of 1731 participants) but also other high-risk genes, surveillance was associated with a substantially higher proportion of stage I detected PDAC compared with PDACs detected outside surveillance (31% vs. 10%) [91]. Correspondingly, 5-year overall survival was higher in the surveillance group (50% vs. 9%). Similarly, a prospective study restricted to carriers of PVs in *CDKN2A* reported higher rates of stage I PDAC detection compared with the general population (38.7% vs. 5.8%). This translated into improved 5-year survival compared to PDAC detected outside surveillance (32.4% vs. 4.3%), even after adjustment for lead-time bias [92].

Collectively, these findings provide some evidence that PDAC surveillance can improve outcomes in selected high-risk populations. However, such benefits have not been demonstrated across all hereditary risk groups and extrapolation to other syndromes, including LS, remains uncertain. Importantly, PDAC surveillance is also associated with potential harms, including overdiagnosis and overtreatment [81]. In some surveillance programs, unnecessary pancreatic surgery has been reported in more than 50% of individuals undergoing intervention [81]. Additionally, little is known about the cost-effectiveness of pancreatic cancer surveillance [93]. Decisions about recommending surveillance should balance its uncertain benefits against the associated burdens and costs. Therefore, currently there is not enough evidence to offer PDAC surveillance in LS as standard of care. Robust prospective evidence is needed before implementation can be recommended.

### Future directions in pancreatic cancer surveillance

Despite evidence supporting the added value of PDAC surveillance in selected high-risk individuals, approximately two-thirds of individuals continue to be diagnosed at a late stage (stage II or higher) [94]. This indicates a need for biomarkers or new methodologies, such as AI-driven techniques, to improve early stage detection.

Currently, carbohydrate antigen 19-9 (CA19-9) is the only biomarker approved by the FDA for PDAC. However, due to its limited diagnostic performance, it is used primarily for disease monitoring rather than for early detection in surveillance settings [95]. Recently, a promising multi-analyte marker (*PancreaSure*) has been externally validated in a cohort including 202 individuals with stage I-II PDAC and 864 controls, demonstrating an AUC of 0.90 (95% CIs 0.87–0.93) [96]. Nonetheless, validation in a true surveillance cohort is still required before clinical implementation.

AI-based approaches in PDAC imaging are promising, however, substantial work remains. A key challenge currently being addressed is the limited quality and quantity of available training data. To overcome this limitation, automated segmentation algorithms are being developed to generate large, high-quality datasets, thereby enabling model training and creating opportunities for integration of AI into surveillance imaging [97].

## Conclusions

In this overview, we demonstrate that LS carriers have an increased risk of gastric, small bowel, and pancreatic cancer. Interpretation of cancer incidence data is challenged by several limitations, including the overall low number of cancer cases and substantial heterogeneity of study populations. In addition, most large cohort studies originate from centers in the United States and Europe, resulting in limited geographic and ethnic diversity and underrepresentation of high-incidence regions. Evidence regarding the effectiveness of UGI surveillance is largely derived from retrospective cohort studies and has thus failed to demonstrate an impact on robust clinical endpoints, such as UGI cancer-related mortality. Given the rarity of these malignancies and the associated logistical challenges, randomized controlled trials are unlikely to be feasible. Consequently, there is a clear need for large, well-designed prospective cohort studies to better define the benefits and limitations of surveillance strategies. The absence of robust efficacy data is reflected in the lack of consensus among current clinical guidelines. With the exception of universal *H. pylori* testing and eradication, recommendations for surveillance of gastric, small bowel, and pancreatic cancer vary considerably across guidelines. To date, there is no universally accepted protocol for UGI endoscopy in LS, and standardized quality metrics comparable to those established for colonoscopy are lacking.

In this article, we provide an overview of emerging surveillance approaches. At present, many of these strategies have not yet been validated in the general population and require further evaluation in large, dedicated LS cohorts. Centralized surveillance in specialized hereditary cancer centers is essential to accommodate the heterogeneity of LS, provide tailored surveillance approaches, and support the generation of high-quality prospective data.

## Data Availability

No datasets were generated or analysed during the current study.
